# Prevalence of deleterious germline variants in risk genes including *BRCA1/2* in consecutive ovarian cancer patients (AGO-TR-1)

**DOI:** 10.1371/journal.pone.0186043

**Published:** 2017-10-20

**Authors:** Philipp Harter, Jan Hauke, Florian Heitz, Alexander Reuss, Stefan Kommoss, Frederik Marmé, André Heimbach, Katharina Prieske, Lisa Richters, Alexander Burges, Guido Neidhardt, Nikolaus de Gregorio, Ahmed El-Balat, Felix Hilpert, Werner Meier, Rainer Kimmig, Karin Kast, Jalid Sehouli, Klaus Baumann, Christian Jackisch, Tjoung-Won Park-Simon, Lars Hanker, Sandra Kröber, Jacobus Pfisterer, Heidrun Gevensleben, Andreas Schnelzer, Dimo Dietrich, Tanja Neunhöffer, Mathias Krockenberger, Sara Y. Brucker, Peter Nürnberg, Holger Thiele, Janine Altmüller, Josefin Lamla, Gabriele Elser, Andreas du Bois, Eric Hahnen, Rita Schmutzler

**Affiliations:** 1 Department of Gynecology & Gynecologic Oncology, Kliniken Essen-Mitte, Essen, Germany; 2 Center for Hereditary Breast and Ovarian Cancer, University Hospital Cologne, Medical Faculty, Cologne, Germany; 3 Center for Integrated Oncology (CIO), University Hospital Cologne, Medical Faculty, Cologne, Germany; 4 Coordinating Center for Clinical Trials, Philipps-University of Marburg, Marburg, Germany; 5 Department of Women’s Health, Tübingen University Hospital, Tübingen, Germany; 6 National Center for Tumor Disease/Department of Gynecology, University of Heidelberg, Heidelberg, Germany; 7 Institute of Human Genetics, University of Bonn, Bonn, Germany; 8 Department of Gynecology and Gynecologic Oncology, University Medical Center Hamburg-Eppendorf, Hamburg, Germany; 9 Department of Gynecology, University Hospital Munich-Großhadern. Munich, Germany; 10 Department of Gynecology, University of Ulm, Ulm, Germany; 11 Department of Gynecology, University of Frankfurt, Frankfurt, Germany; 12 Onkologisches Therapiezentrum, Krankenhaus Jerusalem, Hamburg, Germany/ Department of Gynecology, University of Kiel, Kiel, Germany; 13 Department of Gynecology, University of Kiel, Kiel, Germany; 14 Department of Gynecology, Evangelisches Krankenhaus, Duesseldorf, Germany; 15 Department of Gynecology, University of Essen, Essen, Germany; 16 Department of Gynecology and Obstetrics, Medical Faculty and University Hospital Carl Gustav Carus, Technische Universität Dresden, Dresden, Germany; 17 National Center for Tumor Diseases (NCT), Partner Site Dresden, Dresden, Germany; 18 German Cancer Consortium (DKTK), Dresden and German Cancer Research Center (DKFZ), Heidelberg, Germany; 19 Department of Gynecology and Gynecological Oncology, Charité, Campus Virchow, Berlin, Germany; 20 Department of Gynecology and Obstetrics, Klinikum Ludwigshafen, Ludwigshafen, Germany; 21 Department of Gynecology, Gynecologic Endocrinology & Oncology, Philipps-University Marburg, Marburg, Germany; 22 Department of Gynecology and Obstetrics, Sana Klinikum, Offenbach, Germany; 23 Department of Gynecology, Medizinische Hochschule Hannover, Hannover, Germany; 24 Department of Gynecology & Obstetrics, University Hospital Schleswig-Holstein, Campus Lübeck, Lübeck, Germany; 25 Center of Gynecological Oncology, Kiel, Germany; 26 Institute of Pathology, University Hospital Bonn, Bonn, Germany; 27 Department of Gynecology, Klinikum rechts der Isar, Technical University of Munich, Munich, Germany; 28 University Hospital Bonn, Department of Otolaryngology, Head and Neck Surgery, Bonn, Germany; 29 Department of Gynecology, Dr. Horst-Schmidt-Kliniken, Wiesbaden, Germany; 30 Department of Gynecology, University of Würzburg, Würzburg, Germany; 31 Center for Molecular Medicine Cologne (CMMC), University of Cologne, Cologne, Germany; 32 Cologne Center for Genomics (CCG), University of Cologne, Cologne, Germany; 33 Cologne Excellence Cluster on Cellular Stress Responses in Aging-Associated Diseases (CECAD), University of Cologne, Cologne, Germany; 34 AGO Study Group, Wiesbaden, Germany; CNR, ITALY

## Abstract

**Background:**

Identification of families at risk for ovarian cancer offers the opportunity to consider prophylactic surgery thus reducing ovarian cancer mortality. So far, identification of potentially affected families in Germany was solely performed via family history and numbers of affected family members with breast or ovarian cancer. However, neither the prevalence of deleterious variants in *BRCA1/2* in ovarian cancer in Germany nor the reliability of family history as trigger for genetic counselling has ever been evaluated.

**Methods:**

Prospective counseling and germline testing of consecutive patients with primary diagnosis or with platinum-sensitive relapse of an invasive epithelial ovarian cancer. Testing included 25 candidate and established risk genes. Among these 25 genes, 16 genes (*ATM*, *BRCA1*, *BRCA2*, *CDH1*, *CHEK2*, *MLH1*, *MSH2*, *MSH6*, *NBN*, *PMS2*, *PTEN*, *PALB2*, *RAD51C*, *RAD51D*, *STK11*, *TP53*) were defined as established cancer risk genes. A positive family history was defined as at least one relative with breast cancer or ovarian cancer or breast cancer in personal history.

**Results:**

In total, we analyzed 523 patients: 281 patients with primary diagnosis of ovarian cancer and 242 patients with relapsed disease. Median age at primary diagnosis was 58 years (range 16–93) and 406 patients (77.6%) had a high-grade serous ovarian cancer. In total, 27.9% of the patients showed at least one deleterious variant in all 25 investigated genes and 26.4% in the defined 16 risk genes. Deleterious variants were most prevalent in the *BRCA1* (15.5%), *BRCA2* (5.5%), *RAD51C* (2.5%) and *PALB2* (1.1%) genes. The prevalence of deleterious variants did not differ significantly between patients at primary diagnosis and relapse. The prevalence of deleterious variants in *BRCA1/2* (and in all 16 risk genes) in patients <60 years was 30.2% (33.2%) versus 10.6% (18.9%) in patients ≥60 years. Family history was positive in 43% of all patients. Patients with a positive family history had a prevalence of deleterious variants of 31.6% (36.0%) versus 11.4% (17.6%) and histologic subtype of high grade serous ovarian cancer versus other showed a prevalence of deleterious variants of 23.2% (29.1%) and 10.2% (14.8%), respectively. Testing only for *BRCA1/2* would miss in our series more than 5% of the patients with a deleterious variant in established risk genes.

**Conclusions:**

26.4% of all patients harbor at least one deleterious variant in established risk genes. The threshold of 10% mutation rate which is accepted for reimbursement by health care providers in Germany was observed in all subgroups analyzed and neither age at primary diagnosis nor histo-type or family history sufficiently enough could identify a subgroup not eligible for genetic counselling and testing. Genetic testing should therefore be offered to every patient with invasive epithelial ovarian cancer and limiting testing to *BRCA1/2* seems to be not sufficient.

## Introduction

Ovarian cancer is the leading cause of death of all gynecologic cancers both in the European Union and the United States [1]. In the US, 22,280 patients were diagnosed in 2016 for ovarian cancer and 14,240 died [2]. Despite improvements in chemotherapy [3,4,5] and surgery [6,7] still most patients relapse and finally die of disease. A decade ago, it was estimated that about 10–15% of all patients have a hereditary risk for ovarian cancer [8,9]. The first identified predisposition genes were *BRCA1* and *BRCA2* with a lifetime penetrance regarding ovarian cancer of 35–59% (*BRCA1*) and 11–17% (*BRCA2*), respectively [10,11,12,13]. Unfortunately, several trials which tried to establish a successful screening for ovarian cancer failed [14]. The development of a serous tubal intraepithelial carcinoma (STIC) as a precursor lesion in the fallopian tube with the potential to metastasize within the peritoneal cavity before a solid tumor in the pelvis can be detected, might be one reason for the trial failures [15,16]. Consequently, the majority of patients is diagnosed at an advanced stage with the above-mentioned mortality. So far, the only effective method to reduce the mortality is risk-reducing surgery including bilateral salpingo-oophorectomy (rrBSO) [17]. As rrBSO cannot be recommend to the general population, identification of population at risk might help to identify those women in whom the benefit of rrBSO may outweight it’s burden. Identifying families with deleterious *BRCA1/2* variants might provide this opportunity.

So far, we did not know how many family members must be affected before we can assume an elevated risk for their relatives and especially, if one affected member already qualifies for genetic testing. A further hurdle is the decreasing number of family members in Germany over decades and more recently the increase of so-called blended families. In Germany and in most other countries, genetic testing of patients with ovarian cancer is limited to patients with a family history of breast and/or ovarian cancer. The aim of this study was to investigate the prevalence of genetic risk factors in consecutive patients with ovarian cancer irrespective of family history and histologic subtype treated in centers of the Arbeitsgemeinschaft Gynäkologische Onkologie (AGO) study group. In addition, we wanted to evaluate patient satisfaction with gynecological oncology counseling.

## Methods

### Patients

The protocol was approved by the ethical committee of the Landesaerztekammer Nordrhein (Nr. 2014340) and registered (NCT02222883); all patients gave written informed consent prior to any study related procedure. Patients aged at least 18 years with primary diagnosis or platinum-sensitive relapse of invasive epithelial ovarian cancer in Germany were tested and counselled in 20 centers of the AGO study group in Germany. Platinum sensitive disease was defined as relapse at least 6 months after prior platinum based chemotherapy. It was allowed to include patients up to 6 months after last platinum dose and patients with more than one prior platinum line. We excluded patients with non-epithelial ovarian malignancy and those with a platinum-free interval of less than 6 months. Demographic data, disease characteristics, family history, and medical history were documented. Further follow up of the patients is planned for 5 years and will be reported later. A positive family history was defined as at least one relative with breast cancer or ovarian cancer or breast cancer in personal history. This means, that also patients with the diagnosis of breast cancer in personal history and now the additional diagnosis of ovarian cancer were classified as having a positive family history.

### Gene selection

Germline testing was centrally performed at the Center of Hereditary Breast and Ovarian Cancer at the University of Cologne, Germany. Testing included 25 established and candidate risk genes related to ovarian and/or breast cancer or rare cancer predisposition syndromes (*ATM*, *BARD1*, *BRCA1*, *BRCA2*, *BRIP1*, *BUB1B*, *CDH1*, *CHEK1*, *CHEK2*, *FAM175A*, *FANCM*, *MLH1*, *MSH2*, *MSH6*, *MRE11A*, *NBN*, *PMS2*, *PTEN*, *PALB2*, *RAD50*, *RAD51C*, *RAD51D*, *STK11*, *TP53*, *XRCC2)*. Among these 25 genes, 16 were defined as as established cancer “risk genes”, including the Lynch syndrome-associated genes (*MLH1*, *MSH2*, *MSH6*, *PMS2*), genes causative for rare cancer predisposition syndromes (*CDH1*, *PTEN*, *STK11*, *TP53)*, and genes known to contribute to hereditary breast and/or ovarian cancer risk, namely *ATM*, *BRCA1*, *BRCA2*, *CHEK2*, *NBN*, *PALB2*, *RAD51C* and *RAD51D* [18,19,20,21,22]. For the remaining 9 genes, only limited evidence is available so far for *BUB1B*, *BARD1*, *BRIP1*, *CHEK1*, *FANCM*, *FAM175A*, *MRE11A*, *RAD50* and *XRCC2* [23,24,25,26,27,28]. *BUB1B* and *CHEK1* were included because deleterious variants in both genes have been described in ovarian cancer patients [29]. We classified the analyzed genes in 3 different categories: *BRCA1/BRCA2* (group A), 16 risk genes (group B) and all 25 genes (group C).

### Genetic analyses

Genomic DNA was isolated from venous blood samples using standard methods. All samples were screened for gross genomic aberrations in the *BRCA1/2* genes by Multiplex Ligation-dependent Probe Amplification (MLPA) using the SALSA^®^ MLPA^®^ probemixes P002 (*BRCA1*) and P045 (*BRCA2*) (MRC-Holland, Amsterdam, The Netherlands) according to the manufacturers protocol. Data was analysed using the Coffalyzer.Net v.140429.1057 software (MRC-Holland). All *BRCA1*/2 deletions/duplications were verified using the SALSA^®^ MLPA^®^ probemixes P087 (*BRCA1*) or P077 (*BRCA2*), respectively (MRC-Holland). All samples were subsequently analyzed by next generation sequencing (NGS) covering the entire coding regions and exon-flanking sequences (±15nt) of the 25 above-mentioned genes. For NGS, we employed a customer-tailored SureSelect gene panel (Agilent, Santa Clare, U.S.) using the XT Target Enrichment for Illumina Paired-End Multiplexed Sequencing protocol optimized for 200ng of genomic DNA (Agilent). Sequencing was performed on MiSeq or HiSeq4000 devices (Illumina, San Diego, U.S.). Bioinformatic analyses were carried out using the VARBANK version 2.10 pipeline of the Cologne Center for Genomics. In addition, the obtained NGS data were utilized to identify LGRs (large genomic rearrangements) in non *BRCA1/2* genes using an *in silico* CNV-Tool incorporated in the Sophia DDM^®^ platform (Sophia Genetics). Conspicuous regions indicative for a duplication or deletion were verified using an appropriate SALSA^®^ MLPA^®^ probemix, if available (MRC-Holland) or by aCGH (Array comparative genomic hybridization) using a customized probe set covering the regions of interest (Agilent).

### Variant classification

Variant classification was performed in accordance with the regulations of the international ENIGMA consortium (Evidence-based Network for the Interpretation of Germline Mutant Alleles; https://enigmaconsortium.org; version 1.1: 26^th^ of March 2015). All genetic variants were classified using a 5-tier variant classification system as proposed by the International Agency for Research on Cancer (IARC) Unclassified Genetic Variants Working Group, namely, deleterious = class 5, likely deleterious = class 4, variant of uncertain significance (VUS) = class 3, likely benign = class 2, and benign = class 1 [30]. Variants reported to occur in large outbred control reference groups at an allele frequency ≥1% (MAF ≥ 0.01) are generally considered to be benign (class 1). Class 4/5 variants are subsequently denominated as ´deleterious variants´. All deleterious variants were validated by Sanger sequencing.

### Counselling

Counselling was performed by gynecologic oncologists according to local standard and we evaluated the patient perspectives and satisfaction regarding testing and counseling by a survey. Patients had a BRCA elucidation before testing by their treating gynecologic oncologist and in case of a positive result a subsequent counselling by a gynecologic oncologist or geneticist depending on the local standard. The patients were asked the following questions after testing:

- Finden Sie es gut, dass Ihnen eine Beratung und Testung bzgl. ihres familiären Risikos angeboten wurde? (Did you appreciate that counselling and testing regarding your family risk was offered to you?)
- Waren Sie mit der Beratung zufrieden? (Were you satisfied with the counselling?)
- Waren Sie mit der Ergebnismitteilung zufrieden? (Were you satisfied with how the result was communicated?)

### Statistical methods

The protocol stated a target sample size of 500 patients. No formal power analysis was performed. The primary objectives were to assess the prevalence of deleterious germline variants in the investigated genes. All analyses are merely descriptive; no confirmatory hypothesis testing was done. The prevalence was calculated as number of patients with at least one deleterious variant in the respective genes or gene categories divided by all tested patients. SAS version 9.4 was used for all statistical analyses. Further details are provided in the study protocol (S1 study protocol and S1 Trend Statement Checklist)

## Results

In total, 525 patients were registered and blood was sent for testing in 523 cases including 281 patients with primary ovarian cancer and 242 with relapsed platinum-sensitive ovarian cancer between 3/2015 and 7/2015 (Table 1). The majority of the patients were of White/Caucasian/European heritage (96.8%) followed by White/Arabic/North African heritage with 1.3%. Other ethnicities were more infrequent (< 0.5%). The mean age at primary diagnosis of all patients was 58 years, 43% had a positive family history and 77.6% showed a high-grade serous ovarian cancer (Table 1). In total, 146 of 523 patients (27.9%) showed a deleterious variant in at least one of the investigated genes (S1 Table), of which 9 patients carried two deleterious germline variants (S2 Table). In the overall sample, 109 patients carried a deleterious variant in *BRCA1*, *BRCA2*, or both genes (Table 2, S1 and S2 Tables). In this subgroub of 109 patients with deleterious *BRCA1/2* variants, 6 carried deleterious variants in further genes investigated in this study (Table 2). Deleterious germline variants in *BRCA1* were most abundant (81 patients, 15.5%), followed by *BRCA2* (29 patients, 5.5%), *RAD51C* (13 patients, 2.5%) and *PALB2* (6 patients, 1.1%). Deleterious variants in all other genes analyzed were identified in less than 1% of the patients each (Table 2). The combined analysis showed the following prevalence of deleterious veriants for the respective categories: *BRCA1/2*: 20.8% (group A, 109 patients); risk genes: 26.4% (group B, 138 patients); all genes: 27.9% (group C, 146 patients). The number of patients with at least one deleterious vatiant in any gene (group C) was slightly though not statistically significantly higher in the relapsed versus the primary diagnosis cohort (21.4% [76 of 242] versus 24.9% [70 of 281]; two-sided chi-square p = 0.1).

**Table 1:** Patient characteristics.

Variable	All (n = 523)
Age (mean, range) [years]	58 (16–93)
Positive family history (%)	225 (43.0)
Histologic subtype (%)	
High grade serous	406 (77.6)
High grade endometrioid	23 (4.4)
Low grade serous	18 (3.4)
Low grade endometrioid	7 (1.3)
Clear cell	6 (1.1)
Mucinous	9 (1.7)
Others/unspecified	45 (8.6)
Missing	9 (1.7)
Timepoint of inclusion	
Primary ovarian cancer	281 (53.7)
Recurrent ovarian cancer	242 (46.3)

**Table 2:** Prevalence of deleterious variants in the selected genes. No deleterious variants were observed in *BARD1*, *CDH1*, *MLH1*, *PMS2*, *PTEN*, *STK11* and *TP53*.

Genes / gene categories	Patients, (% of all; n = 523)
*BRCA1/2* (group A)	109 (20.8)
risk genes (group B)	138 (26.4)
any gene (group C)	146 (27.9)
**Group A (109 patients)**	
*BRCA1* only	78 (15.0)
*BRCA1* and *NBN*	1 (0.2)
*BRCA1* and *XRCC2*	1 (0.2)
*BRCA1* and *BRCA2*	1 (0.2)
*BRCA2* only	24 (4.6)
*BRCA2* and *FANCM*	2 (0.4)
*BRCA2* and *RAD50*	1 (0.2)
*BRCA2* and *BUB1B*	1 (0.2)
**Group B (138 patients)**	
*BRCA1/2* carriers	109 (20.8)
*RAD51C* only	13 (2.5)
*PALB2* only	5 (1.0)
*PALB2* and *ATM*	1 (0.2)
*ATM* only	1 (0.2)
*RAD51D* only	3 (0.6)
*MSH2* only	2 (0.4)
*CHEK2* and *BRIP1*	1 (0.2)
*CHEK2* only	1 (0.2)
*NBN* only	1 (0.2)
*MSH6* only	1 (0.2)
**Group C (146 patients)**	
risk genes (group B)	138 (25.8)
*FANCM* only	3 (0.6)
*MRE11A* only	2 (0.4)
*CHEK1* only	1 (0.2)
*FAM175A* only	1 (0.2)
*BRIP1* only	1 (0.2)

Furthermore, we analyzed the prevalence of deleterious variants by different subgroups regarding age (< versus ≥ 60 years), family history (positive versus negative) and histologic subtypes (high-grade serous versus others). All analyses in all subgroups showed a prevalence of deleterious *BRCA1/2* variants above 10.0%. The highest prevalence of deleterious *BRCA1/2* variants was 30.2% in patients below the age of 60 years at primary diagnosis and 31.6% with a positive family history, respectively. Of note, 33/109 patients (30.3%) with deleterious *BRCA1/2* variants would have been missed by using the classical criteria family history. This rate increases to missing 51/138 patients (37%) regarding the 16 risk genes as defined in our series. However, the threshold frequency of 10% that qualifies for reimbursement of consultation and testing was observed in every subgroup analyzed and even analysis per decade of patients’ age did not identify any subgroup with a lower rate of deleterious variants—unfortunately, we were not able to analyze patients in their octogenarium who are underrepresented in our trial with 11 patients only.

The prevalence of deleterious variants in the 16 risk genes ranged from 14.8% (patients with a histologic subtype other than HGS, Table 3) to 36.0% (patients with a positive family history, Table 3) and the prevalence of deleterious variants in any of the investigated genes from 16.7 (patients with a histologic subtype other than HGS, Table 3) to 36.4% (patients with a positive family history, Table 3). The number of patients with non high-grade serous histologic subtypes was limited, however, deleterious *BRCA1/2* variants were also detected in non high grade serous histo-types. Twenty-three patients had high-grade endometrioid histology. This subtype showed a prevalence of deleterious variants in the *BRCA1/2* genes of 13%, 21.7% had a deleterious variant in at least one of the 16 risk genes. Furthermore, we had 18 patients with low-grade serous histology (deleterious variant in *BRCA1/2*: 5.6%, risk gene: 11.1%, any gene: 16.7%). All other known histologic subtypes included less than 10 patients (Table 4). The rate of deleterious variants in the Lynch-associated genes (*MSH2*, *MSH6*, *MLH1*, *PMS2*) was 0.6% (3 patients; 2 *MSH2*-positive patients, 1 with high grade serous and 1 with high-grade endometrial subtype; 1 *MSH6*-positive patient with high-grade serous subtype).

**Table 3:** Prevalence of deleterious variants within subgroups.

Subgroup	n	*BRCA1/2* positive n (%)	risk genes n (%)	any gene n (%)
All (%)	523	109 (20.8)	138 (26.4)	146 (27.9)
Age*				
< 60 years	268	81 (30.2)	89 (33.2)	94 (35.1)
≥ 60 years	254	27 (10.6)	48 (18.9)	51 (20.1)
Family history				
Positive	225	71 (31.6)	81 (36.0)	82 (36.4)
Negative	289	33 (11.4)	51 (17.6)	58 (20.1)
Histologic subtype*				
High grade serous (HGS)	406	94 (23.2)	118 (29.1)	123 (30.3)
other	108	11 (10.2)	16 (14.8)	18 (16.7)
Within subset of HGS				
Primary diagnosis	203	46 (22.7)	54 (26.6)	57 (28.1)
Relapse	203	48 (23.6)	64 (31.5)	66 (32.5)

* Age class was missing for 1 patient; histologic subtype was missing for 9 patients.

**Table 4:** Histologic subtypes and prevalence of deleterious variants in patients with serous, endometrioid, mucinous or clear cell ovarian cancer.

Histologic subtype	n	*BRCA1/2* positive n (%)	risk gene positive n (%)	any gene n (%)
High grade serous	406	94 (23.2)	118 (29.1)	123 (30.3)
Low grade serous	18	1 (5.6)	2 (11.1)	3 (16.7)
High grade endometrioid	23	3 (13.0)	5 (21.7)	5 (21.7)
Low grade endometrioid	7	1 (14.3)	1 (14.3)	1 (14.3)
Mucinous	9	0	0	1 (11.11)
Clear cell	6	0	0	0

The compliance with the survey was high and the rate of responders for each of the questions was 80.9% each (“Did you appreciate that counselling and testing regarding your family risk was offered to you?” and “Were you satisfied with the counselling?”) and 66.2% (“Were you satisfied with how the result was communicated?”). Question 1 (Did you appreciate that counselling and testing regarding your family risk was offered to you?) was answered with “yes” by 98.4%, question 2 (Were you satisfied with the counselling?) by 93.9% and question 3 (Were you satisfied with how the result was communicated?) by 97.4% of the participating patients. The corresponding rates for “No” were 0.5%, 3.2%, and 1.2%, respectively. The rates of “I don’t know” were 1.2%, 2.9% and 1.2%, respectively.

## Discussion

Our data indicate that a genetic background regarding the development of ovarian cancer is present in a higher proportion of patients than anticipated in the past. The strength of our trial is the testing of consecutive unselected patients with ovarian cancer in a multicenter setting. The rate of patients with deleterious variants in *BRCA1/2* was about 21% without any meaningful difference between patients with first diagnosis or relapsed disease. This is in line with other recent publications reporting a rate of *BRCA1/2* carriers of about 16–19% [23, 31,32]. One of the main questions for the future is therefore, how we could prevent about 1/5 of all ovarian cancer cases. According to the National Institute for Health and Care Excellence (NICE), genetic *BRCA1/2* testing is generally recommended when the combined probability to detect a deleterious *BRCA1* and *BRCA2* variant is ≥ 10% [27]. Thus, age at onset and family history were the main selection criteria to identify suitable index patients for genetic testing to identify families at risk. However, this resulted in multiple different guidelines regarding selection criteria differing between the countries and medical societies [33]. Family history and age at onset are only of limited value to identify patients at genetic risk. This was already reported by other authors [34,35,36,37,38]. Using traditional criteria might miss about one third of all patients with a hereditary risk regarding *BRCA1/2*. In our cohort, 33/109 patients (30.28%) with deleterious variants in *BRCA1/2* would have been missed by using only classical criteria like family history. This rate increases to missing 51/138 patients (37%) regarding the 16 risk genes as defined in our series. Disease modifiers that are independent from *BRCA1/2*, *de novo* mutations, or limited family structures may explain the finding that a subgroup of *BRCA1/2*-positive patients with ovarian cancer did not show a positive family history.

A limitation of our series is the limited number of patients with other histologic subtypes than high-grade serous ovarian cancer. Unfortunately, we have only less than 10 patients with a mucinous or clear cell histologic subtype. Therefore, we are not able to give an appropriate answer regarding the rate of deleterious variants in this subtype. Of note, we reported already the finding of deleterious *BRCA1/2* variants in patients with clear cell and mucinous ovarian cancer within an international multicenter first line therapy trial [31]. Whether this finding was by chance or revealed indeed the driver alterations for ovarian cancers remains an open question. In addition, the changed classification of the histologic subtypes in ovarian cancer in 2014 has to be kept in mind as further potential factor. Within a large multicenter study, it was shown that reviewing the pathologic samples by an expert gynecologic pathologist using the old and new classification system results in diagnosing the same histologic subtype again in only 54% [39]. In accordance to another recent presentation, we could also show that gynecologic oncologist led testing shows a high level of satisfaction in patients with ovarian cancer [40].

In conclusion, the limited value of any supporting instruments to identify families at risk of ovarian cancer highlights the necessity to offer germline testing to all patients with ovarian cancer. This is so far the only option we have, to identify families at risk for the usually fatal course of the disease of ovarian cancer. Offering prophylactic surgery in *BRCA1/2*-positive women is the only effective option to decrease ovarian cancer mortality. Therefore, Scotland established offering genetic testing to all patients with non-mucinous histologies of ovarian cancer already in 2013 [41]. The results of our study were already discussed with German health care providers and the Medizinische Dienst der Krankenkassen (MDK), which led to offering testing to all patients with ovarian cancer up to the age of 80 years who were covered by the Verband der Ersatzkassen in Germany in October 2016. Negotiations with further health care providers are ongoing.

## Supporting information

S1 Study protocol (PDF)

S1 Trend Statement Checklist (PDF)

S1 Table Deleterious heterozygous germline variants. (DOCX)

S2 Table Patients carrying two deleterious variants. (DOCX)
