# Supplementary material for: Prevalence of deleterious germline variants in risk genes including BRCA1/2 in consecutive ovarian cancer patients (AGO-TR-1)
Source: PLoS One. 2017 Oct 20;12(10):e0186043. doi: 10.1371/journal.pone.0186043 (PMC5650145; doi:10.1371/journal.pone.0186043)
Supplement: S1 Study protocol — (PDF) [file pone.0186043.s001.pdf]

INCIDENCE OF BRCA IN PATIENTS WITH PRIMARY OR PLATINUM SENSITIVE RECURRENT OVARIAN CANCER

Protocol ID:

AGO-TR 1

(BRCA-Screening)

A Project of the AGO Study Group

AGO Study Group
Kaiser-Friedrich-Ring 71
65185 Wiesbaden
Germany

Version V01F / 11-Aug-2014

In Cooperation with
Center for Hereditary Breast and Ovarian Cancer
University of Cologne, Germany

Coordinating Investigator for the AGO Study Group

Philipp Harter, MD
Klinik für Gynäkologie & Gynäkologische Onkologie
Kliniken Essen - Mitte
Evang. Huyssens Stiftung/Knappschaft GmbH
Henricistraße 92
45136 Essen, Germany

Study offices

AGO Study Group

Kaiser-Friedrich-Ring 71
65185 Wiesbaden, Germany

Moltkeplatz 63
45138 Essen, Germany

Biometrics

Alexander Reuß
Koordinierungszentrum für Klinische Studien (KKS) Marburg
Philipps-Universität Marburg
Karl-von-Frisch-Straße 4
35043 Marburg, Germany

Data Management

Koordinierungszentrum für Klinische Studien (KKS) Marburg
Philipps-Universität Marburg
Karl-von-Frisch-Straße. 4
35043 Marburg, Germany

Monitoring

Schantl Pharma Service GmbH
Herderstraße 16
65185 Wiesbaden, Germany

Persons in charge of study
Coordinating Investigator for the AGO Study Group

11.8.14

Philipp Harter, MD Date

Klinik für Gynäkologie & Gynäkologische Onkologie
Kliniken Essen - Mitte
Evang. Huyssens Stiftung/Knappschaft GmbH
Henricistraße 92
45136 Essen, Germany

Biometrics:

11.08.2014

Alexander Reuß Date

Koordinierungszentrum für Klinische Studien Marburg
Philipps-Universität Marburg
Karl-von-Frisch-Straße 4
35043 Marburg, Germany

Table of Contents

1 **Protocol authorization** ... 6

2 **Summary** ... 7

3 **Introduction** ... 10

3.1 Ovarian Cancer and its Treatment ... 10

3.2 BRCA mutation in Ovarian Cancer ... 10

4 **Rationale** ... 11

5 **Objectives** ... 11

6 **Study Design** ... 12

6.1 Patient selection ... 12

6.2 Patients' registration ... 12

6.3 Patients' evaluation ... 12

6.3.1 Baseline Evaluation ... 12
6.3.2 Firstline Treatment Evaluation ... 12
6.3.3 Further Treatment Evaluation ... 13
6.3.4 Follow-up Evaluation ... 13

6.4 Duration of Study ... 13

7 **Sample Collection and Procedures** ... 13

7.1 Archival Tumor Samples ... 13

7.2 Blood Sampling ... 13

7.3 Sample Shipment ... 14

8 **Determination of Safety** ... 14

9 **Statistical Considerations** ... 14

10 **Data Management** ... 14

10.1 Patient Identification List and Patient ID ... 14

10.2 Data entry and documentation ... 14

10.3 Storage of study documents ... 15

11 **Quality assurance** ... 15

12 **Ethical principles** ... 15

12.1 Ethics committee ... 15

12.2 Informing patients ... 16

12.3 Informed consent to participation ... 16

12.4 Use, storage and transmission of data ... 16

13 Legal and administrative regulations 16

13.1 Financing 16

13.2 Patient insurance 16

13.3 Final report and publication 16

13.4 Adherence to protocol and protocol amendments 16

14 List of Abbreviations 17

15 References 18

1 Protocol authorization

**Study title**	INCIDENCE OF BRCA IN PATIENTS WITH PRIMARY OR PLATINUM SENSITIVE RECURRENT OVARIAN CANCER.<br>AGO-TR 1 / BRCA-SCREENING
**Version**	V01F 11-Aug-2014

Principal Investigator

____
place, date

____
signature

____
name (in block letters)

Declaration

With my above-given signature I confirm that the present study is performed in agreement with the most recent version of the declaration of Helsinki and "ICH Guideline on Good Clinical Practice".

2 Summary

TITEL	BRCA-Screening in Ovarian Cancer<br>Incidence of BRCA in patients with primary or platinum sensitive recurrent ovarian cancer.<br>(Protocol ID: AGO-TR 1)
SPONSOR	AGO Research GmbH, AGO Study Group
COOPERATION	Center for Hereditary Breast and Ovarian Cancer, University of Cologne, Germany
PROJECT LEAD	Philipp Harter, MD, Essen, Germany
INDICATION	Primary diagnosis or platinum-sensitive recurrence in patients with epithelial invasive ovarian cancer.
RATIONALE	About 15% of patients with first diagnosis of ovarian cancer are estimated to have a germline mutation regarding BRCA1/2. Whilst BRCA-status was in the past a prognostic factor regarding own outcome of existing breast and/or ovarian cancer and risk regarding secondary malignancy (including potential consequences for relatives) it has now changed to a predictive marker regarding treatment with PARP-inhibitors like Olaparib (*Ledermann et al. New Engl J Med 2012, Ledermann et al. J Clin Oncol 2013*). Unfortunately, details of the incidence of BRCA positive patients with first diagnosis and platinum sensitive recurrent ovarian cancer in the German population are not clearly defined. In addition, germline testing includes intensive counseling of the patient including their families. It might also be feasible to test paraffin embedded tumor regarding somatic BRCA mutations to define responsiveness regarding PARP-inhibition.<br><br>The aim of this prospective registration and translational research study is to evaluate the incidence of BRCA regarding germline and somatic mutations. In addition, we plan to collect tumor samples of first diagnosis and relapse (if available) to look for potential changes in the BRCA-status of the tumor.
STUDY DESIGN	Prospective, non-interventional, national, multi-center study.
NUMBER OF PATIENTS	500 consecutive patients (up to 250 +/-10% patients with primary diagnosis and up to 250 +/- 10% patients with platinum-sensitive recurrence of ovarian cancer)
TARGET POPULATION	Adult female patients with first diagnosed or platinum-sensitive recurrent epithelial invasive ovarian cancer.

OBJECTIVES	**Primary objective:**<br>• Evidence of germline alterations in BRCA1/2 and other ovarian cancer predisposing genes.<br>**Secondary objectives:**<br>• Immunohistochemistry and DNA extraction from tumor samples<br>• Evidence of somatic alterations in BRCA1/2 and other ovarian cancer predisposing genes<br>• Evidence for a BRCAness tumor phenotype in ovarian cancer<br>• Differences of tumor samples from primary and relapsed disease<br>• Evaluation of patient perspectives and satisfaction regarding testing and counseling (survey)<br>**Translational objectives:**<br>• Validate the results from Pennington et al. *(Pennington et al. Clin Cancer Res 2014)*<br>• Evaluate predictive value of PARP-1 expression for HRD mutations<br>• Identify BRCA 1/2 promoter hypermethylation / epigenetic alterations in addition to genetic alterations Identify large rearrangements/recurrent genomic alterations via genome-wide arrayCGH analysis to describe BCRAness tumor phenotype and identify further genes<br>• Identify and characterize large rearrangements in BRCA1/2 with MLPA in the tumor tissue probes
INCLUSION CRITERIA	1. Female ovarian cancer patients aged ≥ 18 years.<br>2. Women with first diagnosis of epithelial ovarian cancer OR women diagnosed with platinum-sensitive recurrent ovarian cancer (i.e., women who have received an initial course of platinum-based chemotherapy and with no evidence of disease progression for at least 6 months after the last administration of a platinum-based regimen for initial treatment).<br>3. Multiple platinum based prior therapies are allowed.
EXCLUSION CRITERIA	1. Non-epithelial ovarian malignancy.<br>2. Platinum resistant or refractory disease.<br>3. Paraffin embedded tumor samples not available.

INTERVENTION	None.
INVESTIGATIONS	Clinical examinations, providing existing tumor samples, blood samples
STUDY DURATION	Start: Q3 2014<br>Recruitment: N = 500 consecutive patients will be included into this study over a period of 6 months.<br>Follow-up: 60 months<br>First Patient In to Last Patient Out: Q3 2014 – Q1 2020
VARIABLES AND OUTCOMES	Demographic:<br>• Year of Birth<br>• Ethnicity<br>Disease Characteristics:<br>• Date of first diagnosis and/or date of recurrence<br>• FIGO Stage, Grading, Histology<br>Medical History:<br>• Personal history of BRCA associated cancer<br>• Family history of BRCA associated cancer<br>• Number of platinum-based chemotherapy lines (if applicable)<br>• Agents, doses, number of cycles , schedules, durations (dates of first and final administration)(if applicable)
	Sample Assessments:<br>• Results of the search for germline alterations in BRCA1/2 and other ovarian cancer predisposing genes<br>• Result of immunohistochemistry<br>• Results of search for somatic alterations in BRCA1/2 and other ovarian cancer predisposing genes<br>• Results of search for BRCAness tumor phenotype in ovarian cancer<br>Outcome:<br>• Progression-free survival (assessment of progression, date of progression)<br>• Overall survival (date of death)
SAMPLE SIZE CALCULATION /<br>STATISTICAL ANALYSIS	The study size is not based on formal statistical considerations. A target study size of at least 500 patients will be considered for the conduct of the study.

3 Introduction

3.1 Ovarian Cancer and its Treatment

Epithelial ovarian cancer and related malignancies represent the fifth most common cause of cancer-related death among women in Europe and the United States of America[1,2]. Ovarian Cancer (OC) alone is the fourth most common cause of cancer-related death in women with estimated 200,000 cases and 125,000 deaths annually worldwide. According to statistics from the Robert-Koch-Institute, in Germany 2010, 7,780 patients were newly diagnosed and 5,599 did from this neoplasm[3].

The incidence of ovarian cancer increases with age and is most prevalent in the eighth decade of life. More than 70% of the patients are diagnosed with advanced disease and less than 40% of women with ovarian cancer are cured[4,5]. Unfortunately, effective screening methods are lacking[6].

The standard therapy for advanced ovarian cancer consists of radical debulking surgery followed by post-operative platinum-based firstline chemotherapy. Since 1996, platinum and paclitaxel combination therapy has become the standard-of-care firstline chemotherapy regimen[7]. Worldwide, the use of carboplatin has replaced that of cisplatin because of carboplatin's superior tolerability profile together with equal effectiveness. However, the success of this approach is limited and approximately 70% of patients fail to achieve complete responses, or eventually relapse, after a varying disease-free interval.

Should a relapse or a progression after firstline therapy occur, cure of the disease is rarely possible. In this case, secondline chemotherapy is usually offered, with a palliative intent. Recurrence-free interval influences the choice of possible secondline chemotherapy, specifically the value of platinum re-exposure[8-10] and is of prognostic importance for the ongoing course of the illness[11]. Patients with ovarian cancer who developed recurrence >6 months after completion of firstline platinum chemotherapy are characterized as having platinum-sensitive disease.

For patients with platinum-sensitive disease, response rates of 20% to 30% may be seen with platinum re-treatment in those with a platinum-free interval if 6 to 12 months. In those patients with treatment-free intervals >12 months, response rates in the range of 30% to 70% may be seen, and some patients may benefit from durable second remissions.

Carboplatin in combination with gemcitabine +/- bevacizumab, pegylated liposomal doxorubicin or paclitaxel represents the main treatment options for patients with relapsed platinum-sensitive disease, being repeated as long as the patients remains platinum sensitive[12].

3.2 BRCA mutation in Ovarian Cancer

An important risk factor for ovarian cancer is genetic predisposition with BRCA1 and BRCA2 mutations (i.e., gBRCAm) which account for the majority of hereditary ovarian cancers. If a lifetime risk for ovarian cancer among women in the general population is estimated to be 1.4%, a woman with BRCA1 or BRCA2 deleterious mutation has a lifetime risk of 15% to 40%[13]. BRCA mutated ovarian cancer patients can also developed ovarian cancer earlier in their life than those without the mutation. Deficiency in BRCA ultimately leads to the accumulation of genetic alterations as a result of the failure of cells arrest and repair DNA damage or to undergo apoptosis, resulting in tumorigenesis. If all ovarian cancer patients underwent gBRCA testing, current estimates indicate that 13% to 14% of the overall ovarian cancer population would have gBRCA1/2 mutations, and the proportion of patients with gBRCA mutations may be as high as 22% in patients with high-grade serous ovarian cancer (HGSOC). In addition, a population of ovarian cancer patient whose tumors harbor BRCA1 and BRCA2 mutations that are not detected in the germline (approx. 7 %) also exist and are defined as somatic BRCA mutations (tBRCAm).

Patients with BRCA-mutated ovarian cancer currently have identical treatment options as sporadic ovarian cancer patients. They seem to have better prognosis compared with the overall relapsed ovarian cancer patient population but the pattern of disease is similar, with patients eventually dying from their disease. Ovarian cancer patients with BRCA mutation represent a small, well defined and medically recognized subpopulation for whom, despite the potential for personalized healthcare, no targeted treatment currently exists.

4 Rationale

About 15% of patients with first diagnosis of ovarian cancer are estimated to have a germline mutation regarding BRCA1/2. Whilst BRCA status was in the past a prognostic factor regarding own outcome of existing breast and/or ovarian cancer and risk regarding secondary malignancy (including potential consequences for relatives) it has now changed to a predictive marker regarding treatment with PARP-inhibitors like Olaparib[14,15]. Unfortunately, details of the incidence of BRCA positive patients with first diagnosis and platinum sensitive recurrent ovarian cancer in the German population are not clearly defined. In addition, germline testing includes intensive counseling of the patient including their families. It might also be feasible to test tumor samples regarding somatic BRCA mutations to define responsiveness regarding PARP-inhibition.

The aim of this prospective registration and translational research study is to evaluate the incidence of BRCA regarding germline and somatic mutations. In addition, we plan to collect tumor samples of first diagnosis and relapse (if available) to look for potential changes in the BRCA-status of the tumor.

5 Objectives

Primary objective:

- Evidence of germline alterations in BRCA1/2 and other ovarian cancer predisposing genes

Secondary objectives:

- Immunohistochemistry and DNA extraction from tumor samples
- Evidence of somatic alterations in BRCA1/2 and other predisposing genes
- Evidence for a BRCAness tumor phenotype in ovarian cancer
- Differences of tumor samples from primary and relapsed disease
- Evaluation of patient perspectives and satisfaction regarding testing and counseling (survey)

Translational objectives:

- Validate the results from Pennington et al.[16]
- Evaluate predictive value of PARP-1 expression for HRD mutations
- Evaluate predictive value of PARP-1 expression for HRD mutations
- Identify BRCA 1/2 promoter hypermethylation / epigenetic alterations in addition to genetic alterations
- Identify large rearrangements/recurrent genomic alterations via genome-wide arrayCGH analysis to describe BCRAness tumor phenotype and identify further genes
- Identify and characterize large rearrangements in BRCA1/2 with MLPA in the tumor tissue probes

6 Study Design

6.1 Patient selection

The patient must fulfill the following inclusion and exclusion criteria.

Inclusion criteria:

- Female ovarian cancer patients aged ≥ 18 years.
- Women with first diagnosis of epithelial ovarian cancer OR women diagnosed with platinum-sensitive recurrent ovarian cancer (i.e., women who have received an initial course of platinum-based chemotherapy and with no evidence of disease progression for at least 6 months after the last administration of a platinum-based regimen for initial treatment).
- Multiple platinum based prior therapies are allowed.

Exclusion criteria:

- Non-epithelial ovarian malignancy.
- Platinum resistant or refractory disease.
- Paraffin embedded tumor samples and slides not available.
-

6.2 Patients' registration

Following the signature of an informed consent and the validation of the inclusion and exclusion assessments, eligible patients will be registered by fax using the CRF for registration. Registration Fax should be send to

KKS Marburg
Philipps University
Karl-von-Frisch-Straße 4
35043 Marburg
Germany

Fax No.: +49 (0) 6421-2866516

6.3 Patients' evaluation

6.3.1 Baseline Evaluation

Patients who have signed the informed consent will undergo the following evaluations

- Age / Ethnicity
- Height / Weight / ECOG
- Medical Gynecological History
- Familial Medical History relevant for BRCA screening
- Lab Values (only total Bilirubin and Creatinine, if not be done in the last 3 months before registration)

6.3.2 Firstline Treatment Evaluation

For patients included with primary diagnosis the following items should be documented

- Tumor Histology
- Firstline Treatment including Maintenance Therapy
- Patient Status

6.3.3 Further Treatment Evaluation

For patients included with platinum-sensitive recurrent disease only the following items should be documented

- Tumor Histology
- Firstline and further Treatments including Maintenance Therapy
- Patient Status

For patients included with primary diagnosis and a platinum-sensitive recurrence occurred data for both statuses should be documented.

6.3.4 Follow-up Evaluation

Follow-up visits will include

- Date death / alive,
- Date of any recurrence / progression,
- New cancer treatment since last visit (yes/no),
- Diagnosis of next malignancy (yes/no).

Follow-up visits should be done every 12 months after date of registration.

6.4 Duration of Study

The study is planned to start Q3 2014 with respect to first patient in (FPI) including a registration period of 6 months. Each patient will be followed 5 years or until death (whichever occurs first).

7 Sample Collection and Procedures

The samples and data from this research will be coded and not labelled with any personal details. Each sample will be identified with the study code and patient enrolment number. In this way biomarker data may be correlated with clinical data, samples destroyed after analysis. However, only the investigator will be able to link the biomarker sample to the individual patient. The samples and any results will remain the responsibility of the sponsor.

The sponsor will not provide biomarker results to patients, their family members, any insurance company, an employer, study investigator, general physician or any third party unless required to do so by law. The patient’s samples will not be used for any other purpose other than those described in the protocol.

The exception to the above is the gBRCA result. This result will be provided to the investigator and will be collected as part of the patient’s demography and medical history details.

7.1 Archived? Tumor Samples

For all enrolled patient, archived tumor samples must be collected for BRCA and further genetic testing. 8 tumor slides should be sent to the University of Cologne, Center for Hereditary Breast and Ovarian Cancer, Germany. Tumor slides should be sending to the University of Cologne during 3 weeks after registration.

7.2 Blood Sampling

After registration 20mL blood collected in EDTA tubes for BRCA and further genetic testing should be send directly after collection to the University of Cologne, Center for Hereditary Breast and Ovarian cancer, Germany.

7.3 Sample Shipment

Each sample (tumor slides and blood) will be send directly after collection to:

Universitätsklinik Köln
Zentrum für Familiären Brust- und Eierstockkrebs
Kerpener Straße 34, Gebäude 47, 8. Etage, Raum 8.016
z. Hd. Frau Sandra Körber / Herr Dr. Jan Hauke
50931 Köln
Germany
Phone: +49 (0) 221 478 86682 or +49 (0) 221 478 98570
Fax: +49 (0) 221 478 87016

8 Determination of Safety

Only spontaneous occurred adverse events in relationship with a substance of AstraZeneca should be documented. These adverse events ("UAWs") should be sending directly to AstraZeneca GmbH, Abteilung für Arzneimittelsicherheit, Tinsdaler Weg 183, 22880 Wedel, Germany.

9 Statistical Considerations

The study size is not based on formal statistical considerations. A target study size of at least 500 patients will be considered for the conduct of the study and is considered representative to answer the study objectives.

No statistical hypothesis applies.

10 Data Management

10.1 Patient Identification List and Patient ID

A patient identification list has to be handled by the investigators. In this list the pseudonymized patient ID is connected with the data to identify the patient unmistakably. The patient identification list is filed in the investigator site file (ISF). The pseudonymized patient ID is combined from the AGO center ID and a sequencing number of the patient starting with 001. This unique patient ID is used for all documentation on electronic case report form (eCRF) and DCF.

10.2 Data entry and documentation

The investigator is responsible for the performance of the study in accordance to ICH GCP guidelines and the study protocol as well as the correct data entry to the corresponding eCRF. The data are documented with the EDC (electronic data capture) system MACRO at KKS Marburg, Germany. The data are entered directly via web browser (Internet Explorer, Firefox or Google Chrome) to the eCRF and are transferred via SSL (128-bit encryption) to the central database. Detailed requirement for using EDC are specified in an EDC Manual.

In order to use the EDC system all staff who are entering and monitoring data are provided with training materials and required documentation by KKS Marburg, Germany. Every person who is using the EDC system has to complete a registration form to confirm that he/she has received the training material and has been trained for data entry. KKS provides a test database for training of data entry.

All clinical data which are collected during the study and the BRCA status have to be documented in the eCRF by authorized person according to the personal log. The participation of the patient in the study has to be documented in the patient record (study title, start and end of study).

The EDC system MACRO has an implemented audit trail. This assures that any documentation and/or changes to database items are traceable anytime. The personal log with signature, name and mnemonic of persons who are authorized to enter data to the eCRF is archived in the investigator site file (ISF) and the trial master file (TMF). Changes and corrections can only be made by persons who are documented in the TMF and who have access to the system with user specific access rights.

Users with monitoring function are not able to enter or change data. They have the possibility to view the data write protected (review function) and they can raise discrepancies and/or SDV marks in case of queries. Discrepancies which appear at data management are forwarded to the responsible monitor.

10.3 Storage of study documents

Originals of all central study documents will be kept in the study office for a period of 10 years after preparation of the publication.

The investigator keeps accrued administrative documents (correspondence with ethic committee, study coordinators, central study office), the patient identification list, signed informed consent and general study documents (protocol, amendments) for the above mentioned period.

Original data of study patients (medical records) must be stored according to the archiving period valid at the respective study site, but not less than 15 years.

11 Quality assurance

In order to guaranty the authenticity and credibility of the data in accordance with ICH GCP, AGO will set up an assurance quality program that includes:

- management of the study according to the AGO SOPs
- quality control of the data provided by the investigator/site is performed by the study monitor whose role is:
    - to match and check the consistency of the data reported in the eCRF with respect to the source documents,
    - to ensure that safety and rights of the study patient are being protected,
    - to ensure that the study is conducted in accordance with the currently approved protocol and any other study agreements and ICH GCP

The investigator and the head of the medical institution (where applicable) agrees to allow the monitor direct access to all relevant documents.

12 Ethical principles

This study is performed in accordance with the most current revision of the Declaration of Helsinki.

12.1 Ethics committee

Study protocol, patient information and informed consent will be submitted each local ethics committee of every participating center. The study will only be started in a center after being approved by the ethics committee.

The co-coordinating investigators will inform the ethics committee about any changes in the study protocol which could interfere with the patient's safety. Furthermore, the committee will be informed of the planned or premature end of the study. The investigators are obliged to consult their competent ethics committee and to wait for approval before including patients into the study, as well as to inform their ethics committee of changes in the protocol and end of study.

12.2 Informing patients

Before enrolment in the study, the treating investigator will inform the patient about the nature of the study, its aims, expected advantages as well as possible risks.

12.3 Informed consent to participation

Each patient must consent in writing to participate in the study. The patient must be given enough time and opportunity to decide on participation and to clarify queries before the beginning of documentation of the study.

The informed consent will be signed by both patient and treating investigator. If the patient is not able to sign herself, oral information and consent have to be confirmed by the signature of a witness.

Layout and text should be adapted according the requirements of local Ethics Committee by the sponsor. . On request, the final forms have to be submitted to the ethics committee for review.
The original document is kept by the investigator, whereas the patient receives a copy.

12.4 Use, storage and transmission of data

Patients will be informed on the fact that their disease-related data will be stored in a pseudonymous form and used for scientific analyses (publications, reports to sponsors). Patients have the right to be informed about stored data. Consent to data access and -transmission will be obtained separately from consent to participation in the study.

13 Legal and administrative regulations

The recommendations of Good Clinical Practice (see ICH-GCP: International Conference on Harmonisation - Good Clinical Practice) in the most recent version are adhered to.

13.1 Financing

This study is supported by AstraZeneca GmbH in Wedel, Germany.

13.2 Patient insurance

Not applicable.

13.3 Final report and publication

After completion of analyses, the coordinating investigator will write a publication.. This report includes the clinical and statistical report as well as conclusions. The study's results will be published irrespective of the nature of the results. The study chairmen, cooperating partners, the statistician and contributing centers will serve as (co)-authors depending absolute number of authors allowed for the respective journal.

13.4 Adherence to protocol and protocol amendments

The study protocol must be thoroughly adhered to. Any deviation must be supported by the investigator and must be documented and justified.

Changes or supplements to the study protocol can only be decided on and authorized by the coordinating principal investigator (amendment).

14 List of Abbreviations

AGO	Arbeitsgemeinschaft Gynaekologische Onkologie
BRCA	Breast Cancer Susceptibility Gene
CGH	Comparative Genomic Hybridization
DCF	Data Clarification Form
DNA	Desoxyribonucleic Acid
ECOG	Eastern Cooperative Oncology Group
eCRF	electronic Case Report Form
EDC	Electronic Data Capture
EDTA	Ethylene Diamine Tetraacetic Acid
FPI	First Patient In
gBRCA(m)	germline BRCA (mutation)
GCP	Good Clinical Practice
HGSOC	high-grade serous Ovarian Cancer
HRD	Homologous Recombination Repair Deficiencies
ICH	International Conference on Harmonization
ID	Identification
ISF	Investigator Site File
KKS	Koordinierungszentrum für Klinische Studien
MLPA	Mutliplex Ligation-dependet Probe
OC	Ovarian Cancer
PARP	Polyadenosine 5'diphosphoribose [poly (ADP ribose)] polymerisation
SDV	Source Date Verification
SOP	Standard Operating Procedures
SSL	Secure Sockets Layer
tBRCAm	Somatic BRCA mutation
TMF	Trial Master File
UAW	Adverse Side Effects / "Unerwuenschte Arzneimittelwirkung"

15 References

1. International Agency for Research on Cancer. Globocan 2012. http://globocan/iarc.fr/. Accessed July 29, 2014.

2. Siegel et al. Cancer statistics, 2011: the impact of eliminating socio-economic and racial disparities on premature cancer deaths. CA Cancer J Clin 2011; 61:212-36.

3. Beiträge zur Gesundheitsberichterstattung des Bundes: Krebs in Deutschland 2009/2010, 9. Ausgabe, 2013, Robert-Koch-Institut und Gesellschaft der epidemiologischen Krebsregister in Deutschland e.V..

4. Fleming et al. Principles and Practice of Gynecological Oncology; 2009:763-836.

5. Jemal et al. Cancer Statistics, 2010. CA Cancer J Clin 2010; 60(5):277-300.

6. Buys et al. Effect of screening on ovarian cancer mortality: the prostate, ling, colorectal and ovarian (PLCO) cancer screening randomized controlled trial. JAMA 2011; 305(22): 2295-303.

7. McGuire et al. Cyclophosphamide and cisplatin compared with paclitaxel and cisplatin in patients with stage III and IV ovarian cancer. N Eng J Med 1996; 334:1-6.

8. Blackledge et al. Response of patients in Phase II studies of chemotherapy in ovarian cancer: implications for patient treatment and the design of Phase II trials. Br J Cancer 1989; 59(4): 650-3.

9. Gore et al. Treatment of relapsed carcinoma of the ovary with cisplatin or carboplatin following initial treatment with these compounds. Gynecol Oncol 1990; 36(2):207-11.

10. Markman et al. Responses to second-line cisplatin-based intraperitoneal therapy in ovarian cancer: influence of a prior response to intravenous cisplatin. J Clin Oncol 1991; 9(10)1801-5.

11. Eisenhauer et al. Predictors of response to subsequent chemotherapy in platinum pre-treated ovarian cancer: a multivariate analysis of 704 patients. Ann Oncol 1997; 8(10): 963-8.

12. Wagner et al. S3-Guideline on diagnostics, therapy and follow-up of malignant ovarian tumours. Geburtshilfe Frauenheilkd 2013; 73(9): 874-89.

13. National Cancer Institute. BRCA1 and BRCA 2: Cancer Risk and Genetic Testing. http://www.cancer.gov/cancertopics/factsheet/Risk/BRCA. Accessed July 29, 2014.

14. Ledermann et al. Olaparib Maintenance Therapy in Platinum-Sensitive Relapsed Ovarian Cancer. N Engl J Med 2012; 366:1382-92.

15. Ledermann et al. Olaparib maintenance therapy in patients with platinum-sensitive Relapsed serous ovarian cancer (SOC) and a BRCA mutation (BRCAm). J Clin Oncol 2013; 31.

16. Pennington et al. Germline and somatic mutations in homologous recombination genes predict platinum response and survival in ovarian, fallopian tube, and peritoneal carcinomas. Clin Cancer Res 2014; 20(3): 764-75.
