# Supplementary material for: Prevalence of deleterious germline variants in risk genes including BRCA1/2 in consecutive ovarian cancer patients (AGO-TR-1)
Source: PLoS One. 2017 Oct 20;12(10):e0186043. doi: 10.1371/journal.pone.0186043 (PMC5650145; doi:10.1371/journal.pone.0186043)
Supplement: S1 Table — (DOCX) [file pone.0186043.s003.docx]

Table S1: Deleterious heterozygous germline variants identified in 523 unselected cases with OC

| gene | NCBI accession | genomic coordinates (hg19) | HGVS (nucleotide)^a^ | HGVS (protein)^b^ | HGMD^c^ | detection method^d^ | no. of patients^f^ |
| --- | --- | --- | --- | --- | --- | --- | --- |
| *ATM* | NM_000051.3 | Chr11:108143509 | c.3214G>T | p.(E1072*) | / | NGS | 1 |
| *ATM* | NM_000051.3 | Chr11:108155008-108155009 | c.3801_3802del(G)2insG | p.(V1268*fs) | / | NGS | 1 |
| *BRCA1* | NM_007294.3 | Chr17:41246432 | c.1116G>A | p.(W372*) | / | NGS | 1 |
| *BRCA1* | NM_007294.3 | Chr17:41246421-41246423 | c.1125_1127del(A)3ins(A)2 | p.(N376Ifs*18) | / | NGS | 1 |
| *BRCA1* | NM_007294.3 | Chr17:41246355 | c.1193C>G | p.(S398*) | CD993415 | NGS | 1 |
| *BRCA1* | NM_007294.3 | / | c.135-?_547+? | / | / | MLPA, CNV-tool | 1 |
| *BRCA1* | NM_007294.3 | Chr17:41245927 | c.1621C>T | p.(Q541*) | / | NGS | 1 |
| *BRCA1* | NM_007294.3 | Chr17:41245861 | c.1687C>T | p.(Q563*) | / | NGS | 3 |
| *BRCA1* | NM_007294.3 | Chr17:41258504 | c.181T>G | p.(C61G) | CM147953 | NGS | 6 |
| *BRCA1* | NM_007294.3 | Chr17:41245674-41245677 | c.1874_1877delTAGTins(TAGT)2 | p.(V627Sfs*4) | / | NGS | 1 |
| *BRCA1* | NM_007294.3 | Chr17:41245505 | c.1902delTins(T)2 | p.(N635*fs) | / | NGS | 1 |
| *BRCA1* | NM_007294.3 | Chr17:41245592-41245595 | c.1953_1956delGAAA | p.(K653Sfs*47) | CI169551 | NGS | 1 |
| *BRCA1* | NM_007294.3 | Chr17:41258473 | c.212G>C | p.(R71T) | CM015252 | NGS | 1 |
| *BRCA1* | NM_007294.3 | Chr17:41256985 | c.213-12A>G | / | / | NGS | 1 |
| *BRCA1* | NM_007294.3 | / | c.-232-?_134+? | / | CM055994 | MLPA, CNV-tool | 1 |
| *BRCA1* | NM_007294.3 | / | c.-232-?_441+? | / | / | MLPA, CNV-tool | 1 |
| *BRCA1* | NM_007294.3 | / | c.-232-?_5074+? | / | CD982819 | MLPA, CNV-tool | 1 |
| *BRCA1* | NM_007294.3 | Chr17:41245210 | c.2338C>T | p.(Q780*) | / | NGS | 2 |
| *BRCA1* | NM_007294.3 | Chr17:41245073 | c.2475delC | p.(D825Efs*21) | CS070398 | NGS | 1 |
| *BRCA1* | NM_007294.3 | Chr17:41245033-41245034 | c.2514_2515del(C)2insC | p.(H839Tfs*7) | CD137601 | NGS | 1 |
| *BRCA1* | NM_007294.3 | Chr17:41244527-41244530 | c.3018_3021delTTCA | p.(H1006Qfs*17) | / | NGS | 1 |
| *BRCA1* | NM_007294.3 | Chr17:41244440-41244444 | c.3104_3108del(T)5ins(T)6 | p.(K1037*fs) | CD100767 | NGS | 1 |
| *BRCA1* | NM_007294.3 | Chr17:41244280 | c.3268C>T | p.(Q1090*) | CD070515 | NGS | 1 |
| *BRCA1* | NM_007294.3 | Chr17:41244057 | c.3481_3491delGAAGATACTAG | p.(E1161Ffs*3) | / | NGS | 3 |
| *BRCA1* | NM_007294.3 | Chr17:41276080 | c.34C>T | p.(Q12*) | / | NGS | 1 |
| *BRCA1* | NM_007294.3 | Chr17:41243844-41243853 | c.3695_3704del(GTAAA)2insGTAAA | p.(V1234Qfs*8) | / | NGS | 2 |
| *BRCA1* | NM_007294.3 | Chr17:41243789 | c.3752_3759del(GTCT)2insGTCT | p.(S1253Rfs*10) | CI102530 | NGS | 2 |
| *BRCA1* | NM_007294.3 | Chr17:41243777-41243780 | c.3768_3771del(AG)2insAG | p.(E1257Gfs*9) | CM160584 | NGS | 1 |
| *BRCA1* | NM_007294.3 | Chr17:41256183-41256184 | c.396_397del(C)2insC | p.(R133Vfs*30) | CI102531 | NGS | 1 |
| *BRCA1* | NM_007294.3 | Chr17:41243455 | c.4093T>G | / | / | NGS | 1 |
| *BRCA1* | NM_007294.3 | Chr17:41234568-41234569 | c.4209_4210del(C)2insC | p.(L1404*) | CS118878 | NGS | 1 |
| *BRCA1* | NM_007294.3 | Chr17:41234556 | c.4222C>T | p.(Q1408*) | / | NGS | 1 |
| *BRCA1* | NM_007294.3 | / | c.442-?_547+? | / | / | MLPA, CNV-tool | 1 |
| *BRCA1* | NM_007294.3 | Chr17:41226489-41226492 | c.4531_4534del(CA)2insCA | p.(H1511Qfs*9) | CS126188 | NGS | 1 |
| *BRCA1* | NM_007294.3 | Chr17:41223242 | c.4689C>G | p.(Y1563*) | CM128411 | NGS | 4 |
| *BRCA1* | NM_007294.3 | Chr17:41222998-41223000 | c.4931_4933del(A)3ins(A)5 | p.(R1645Kfs*14) | CD159809 | NGS | 2 |
| *BRCA1* | NM_007294.3 | Chr17:41222995-41222997 | c.4934_4936del(G)3ins(G)2 | p.(V1646Sfs*12) | / | NGS | 1 |
| *BRCA1* | NM_007294.3 | Chr17:41222949 | c.4964_4982delCTGGCCTGACCCCAGAAGA | p.(S1655Yfs*16) | / | NGS | 1 |
| *BRCA1* | NM_007294.3 | Chr17:41219625 | c.5074G>C | p.(D1692H) | / | NGS | 1 |
| *BRCA1* | NM_007294.3 | / | c.5075-?_5193+? | / | / | MLPA, CNV-tool | 1 |
| *BRCA1* | NM_007294.3 | Chr17:41215958-41215960 | c.5083_5085del(T)3insT | p.(F1695Cfs*3) | CD993415 | NGS | 1 |
| *BRCA1* | NM_007294.3 | Chr17:41215947 | c.5096G>A | p.(R1699Q) | / | NGS | 1 |
| *BRCA1* | NM_007294.3 | Chr17:41215392 | c.5153-2delA | / | / | NGS | 1 |
| *BRCA1* | NM_007294.3 | Chr17:41215363-41215370 | c.5173_5180del(GAAA)2insGAAA | p.(R1726Kfs*3) | / | NGS | 1 |
| *BRCA1* | NM_007294.3 | Chr17:41215349 | c.5193+1delG | / | CM147953 | NGS | 1 |
| *BRCA1* | NM_007294.3 | Chr17:41209082 | c.5264_5266del(C)3ins(C)4 | p.(Q1756Pfs*74) | / | NGS | 13 |
| *BRCA1* | NM_007294.3 | Chr17:41199683 | c.5444G>A | p.(W1815*) | / | NGS | 1 |
| *BRCA1* | NM_007294.3 | / | c.5468-?_5592+? | / | CI169551 | MLPA, CNV-tool | 1 |
| *BRCA1* | NM_007294.3 | / | c.548-?_4185+? | / | CM015252 | MLPA, CNV-tool | 1 |
| *BRCA1* | NM_007294.3 | Chr17:41197784 | c.5503C>T | p.(R1835*) | / | NGS | 1 |
| *BRCA1* | NM_007294.3 | Chr17:41276045 | c.66_69del(AG)2insAG | p.(E23Vfs*17) | CM055994 | NGS | 1 |
| *BRCA1* | NM_007294.3 | Chr17:41276048 | c.66delAins(A)2 | p.(E23Rfs*18) | / | NGS | 1 |
| *BRCA1* | NM_007294.3 | / | c.81-?_5193+? | / | CD982819 | MLPA, CNV-tool | 1 |
| *BRCA1* | NM_007294.3 | / | c.952_1015del64 | p.(H318Rfs*2) | / | NGS | 2 |
| *BRCA2* | NM_000059.3 | Chr13:32907112-32907114 | c.1497_1499del(G)3ins(G)2 | p.(G500Vfs*9) | CS070398 | NGS | 1 |
| *BRCA2* | NM_000059.3 | Chr13:32907419-32907420 | c.1804_1805del(G)2insG | p.(G602Efs*12) | CD137601 | NGS | 1 |
| *BRCA2* | NM_000059.3 | Chr13:32910890 | c.2398_2399del(G)2ins(G)3 | p.(N801*fs) | / | NGS | 1 |
| *BRCA2* | NM_000059.3 | Chr13:32911322 | c.2830A>T | p.(K944*) | CD100767 | NGS | 1 |
| *BRCA2* | NM_000059.3 | Chr13:32911372 | c.2880delG | p.(K960Nfs*4) | CD070515 | NGS | 1 |
| *BRCA2* | NM_000059.3 | Chr13:32911756 | c.3264delTins(T)2 | p.(Q1089Sfs*10) | / | NGS | 1 |
| *BRCA2* | NM_000059.3 | Chr13:32912338-32912339 | c.3846_3847delTG | p.(V1283Kfs*2) | / | NGS | 2 |
| *BRCA2* | NM_000059.3 | Chr13:32912941 | c.4449delA | p.(D1484Tfs*2) | / | NGS | 2 |
| *BRCA2* | NM_000059.3 | Chr13:32913457 | c.4965C>G | p.(Y1655*) | CI102530 | NGS | 1 |
| *BRCA2* | NM_000059.3 | Chr13:32913830 | c.5338delG | p.(E1780Kfs*11) | CM160584 | NGS | 1 |
| *BRCA2* | NM_000059.3 | Chr13:32913988 | c.5496delTins(T)2 | p.(N1833*) | CI102531 | NGS | 1 |
| *BRCA2* | NM_000059.3 | Chr13:32914137 | c.5645C>A | p.(S1882*) | / | NGS | 1 |
| *BRCA2* | NM_000059.3 | Chr13:32914174 | c.5682C>G | p.(Y1894*) | CS118878 | NGS | 2 |
| *BRCA2* | NM_000059.3 | Chr13:32914210-32914215 | c.5718_5723del(CT)3ins(CT)2 | p.(L1908Rfs*2) | / | NGS | 1 |
| *BRCA2* | NM_000059.3 | Chr13:32914438 | c.5946delT | p.(S1982Rfs*22) | / | NGS | 2 |
| *BRCA2* | NM_000059.3 | Chr13:32914759-32914761 | c.6267_6269delGCAinsC | p.(E2089Dfs*2) | CS126188 | NGS | 1 |
| *BRCA2* | NM_000059.3 | Chr13:32914767-32914768 | c.6275_6276del(T)2 | p.(L2092Pfs*7) | CM128411 | NGS | 1 |
| *BRCA2* | NM_000059.3 | Chr13:32920978 | c.6952C>T | p.(R2318*) | CD159809 | NGS | 1 |
| *BRCA2* | NM_000059.3 | Chr13:32930687 | c.7558C>T | p.(R2520*) | / | NGS | 1 |
| *BRCA2* | NM_000059.3 | Chr13:32905141-32905145 | c.767_771delCAAAT | p.(N257Kfs*17) | / | NGS | 1 |
| *BRCA2* | NM_000059.3 | Chr13:32936731 | c.7877G>A | p.(W2626*) | / | NGS | 1 |
| *BRCA2* | NM_000059.3 | Chr13:32936830 | c.7976G>A | p.(R2659K) | / | NGS | 2 |
| *BRCA2* | NM_000059.3 | Chr13:32954273-32954279 | c.9247_9253del(A)7ins(A)8 | p.(T3085Nfs*26) | CD993415 | NGS | 1 |
| *BRCA2* | NM_000059.3 | Chr13:32972316 | c.9666delT | p.(C3222Wfs*27) | / | NGS | 1 |
| *BRIP1* | NM_032043.2 | Chr17:59858254 | c.1741C>T | p.(R581*) | / | NGS | 1 |
| *BRIP1* | NM_032043.2 | Chr17:59820496 | c.2258-1G>A | / | / | NGS | 1 |
| *BUB1B* | NM_001211.5 | Chr15:40512778 | c.2971G>T | p.(E991*) | CM147953 | NGS | 1 |
| *CHEK1* | NM_001114121.2 | Chr11:125513995-125513998 | c.933_936del(TG)2insTG | p.(V312Efs*24) | / | NGS | 1 |
| *CHEK2* | NM_007194.3 | Chr22:29091857 | c.1100delC | p.(T367Mfs*15) | / | NGS | 1 |
| *CHEK2* | NM_007194.3 | Chr22:29130625 | c.85C>T | p.(Q29*) | CI169551 | NGS | 1 |
| *FAM175A* | NM_139076.2 | Chr4:84383709-84383711 | c.1141_1143del(A)3ins(A)4 | p.(A382Sfs*11) | CM015252 | NGS | 1 |
| *FANCM* | NM_020937.3 | Chr14:45645961 | c.4003_4004insG | p.(K1335Rfs*9) | / | NGS | 1 |
| *FANCM* | NM_020937.3 | Chr14:45658326 | c.5101C>T | p.(Q1701*) | CM055994 | NGS | 2 |
| *FANCM* | NM_020937.3 | Chr14:45665687 | c.5653C>T | p.(Q1885*) | / | NGS | 1 |
| *FANCM* | NM_020937.3 | / | c.5717-?_6147+? | p.(G1906_I2048delins46) | CD982819 | aCGH, CNV-tool | 1 |
| *MRE11A* | NM_005591.3 | Chr11:94197282 | c.1217_1222del(A)6ins(A)7 | p.(T408Nfs*49) | / | NGS | 1 |
| *MRE11A* | NM_005591.3 | Chr11:94180454 | c.1714C>T | p.(R572*) | CS070398 | NGS | 1 |
| *MSH2* | NM_000251.2 | Chr2:47705468-47705467 | c.2267_2268insGTAG | p.(Y757*) | CD137601 | NGS | 1 |
| *MSH2* | NM_000251.2 | Chr2:47637374 | c.508C>T | p.(Q170*) | / | NGS | 1 |
| *MSH6* | NM_000179.2 | Chr2:48018160-48018161 | c.355_356del(T)2ins(T)3 | p.(I120Hfs*16) | CD100767 | NGS | 1 |
| *NBN* | NM_002485.4 | Chr8:90983442-90983446 | c.657_661delACAAA | p.(K219Nfs*16) | CD070515 | NGS | 2 |
| *PALB2* | NM_024675.3 | Chr16:23641178-23641179 | c.2296_2297delTC | p.(V767Lfs*5) | / | NGS | 1 |
| *PALB2* | NM_024675.3 | Chr16:23632678 | c.3113+5G>C | / | / | NGS | 1 |
| *PALB2* | NM_024675.3 | Chr16:23614979-23614980 | c.3361_3362del(G)2insG | p.(G1121Vfs*3) | / | NGS | 1 |
| *PALB2* | NM_024675.3 | Chr16:23614900 | c.3441T>A | p.(C1147*) | CI102530 | NGS | 1 |
| *PALB2* | NM_024675.3 | Chr16:23647357-23647358 | c.509_510delGA | p.(R170Ifs*14) | CM160584 | NGS | 1 |
| *PALB2* | NM_024675.3 | Chr16:23647109-23647112 | c.755_758del(CT)2insCT | p.(L253Ifs*3) | CI102531 | NGS | 1 |
| *RAD50* | NM_005732.3 | Chr5:131930560 | c.1794-1G>T | / | / | NGS | 1 |
| *RAD51C* | NM_058216.3 | Chr17:56809906 | c.1026+1G>C | / | CS118878 | NGS | 1 |
| *RAD51C* | NM_058216.3 | Chr17:56772345 | c.199G>T | p.(E67*) | / | NGS | 1 |
| *RAD51C* | NM_058216.3 | Chr17:56772370 | c.224delAins(A)2 | p.(Y75*) | / | NGS | 4 |
| *RAD51C* | NM_058216.3 | Chr17:56774151 | c.502A>T | p.(R168*) | CS126188 | NGS | 1 |
| *RAD51C* | NM_058216.3 | Chr17:56774173-56774174 | c.524_525del(C)2ins(C)3 | p.(C176Lfs*27) | CM128411 | NGS | 1 |
| *RAD51C* | NM_058216.3 | / | c.706-?_837+? | p.(R237_V280del) | CD159809 | MLPA, CNV-tool | 1 |
| *RAD51C* | NM_058216.3 | Chr17:56787218 | c.706-2A>G | / | / | NGS | 2 |
| *RAD51C* | NM_058216.3 | / | c.706-4423_1131+7851 | / | / | MLPA, CNV-tool | 2 |
| *RAD51D* | NM_002878.3 | Chr17:33434127-33434130 | c.357_360delTATG | p.(C119Wfs*16) | / | NGS | 1 |
| *RAD51D* | NM_002878.3 | Chr17:33433404 | c.576+1G>A | / | / | NGS | 1 |
| *RAD51D* | NM_002878.3 | Chr17:33430317 | c.694C>T | p.(R232*) | CD993415 | NGS | 1 |
| *XRCC2* | NM_005431.1 | Chr7:152357811-152357813 | c.94_96del(T)3ins(T)2 | p.(F32Lfs*30) | / | NGS | 1 |

Legend to table S1: All 523 DNA samples were subjected to next generation sequencing (NGS) and screened for deleterious *BRCA1* and *BRCA2* variants by MLPA. NGS analysis revealed 43 different deleterious *BRCA1* variants in 72 patients and 24 different deleterious *BRCA2* variants in 29 patients. MLPA revealed a total of 9 different heterozygous large genomic *BRCA1* rearrangements in 9 patients. In addition, NGS analysis revealed heterozygous deleterious variants in 23 additional genes (*ATM, BARD1, BRIP1, BUB1B, CDH1, CHEK1, CHEK2, FAM175A, FANCM, MLH1, MSH2, MSH6, MRE11A, NBN, PMS2, PTEN, PALB2, RAD50, RAD51C, RAD51D, STK11, TP53, XRCC2*). Bioinformatic analyses of NGS-data (CNV-tool) identified 3 different large genomic rearrangements (1x*FANCM*, 2x*RAD51C*) in 4 patients which were verified by MLPA or aCGH (data not shown). For each deleterious variant, the consequences on nucleotide and protein level according to the HGVS nomenclature (a, b), the HGMD accession number (c), detection method (d) and number of mutation carriers (f) and patient IDs are given (g). Patients with deleterious mutations in more than one gene are listed in table S2
