# Supplementary material for: Prevalence of deleterious germline variants in risk genes including BRCA1/2 in consecutive ovarian cancer patients (AGO-TR-1)
Source: PLoS One. 2017 Oct 20;12(10):e0186043. doi: 10.1371/journal.pone.0186043 (PMC5650145; doi:10.1371/journal.pone.0186043)
Supplement: S2 Table — (DOCX) [file pone.0186043.s004.docx]

Table S2: Patients carrying two deleterious varians

| patient ID | Gene | genomic coordinates (hg19) | HGVS (nucleotide)^a^ | HGVS (protein)^b^ | HGMD^c^ | detection method^d^ |
| --- | --- | --- | --- | --- | --- | --- |
| 1 | *ATM* | Chr11:108155008-108155009 | c.3801_3802del(G)2insG | p.V1268*fs | CD961800 | NGS |
|  | *PALB2* | Chr16:23647109-23647112 | c.755_758del(CT)2insCT | p.L253Ifs*3 | CD070515 | NGS |
| 2 | *BRIP1* | Chr17:59820496 | c.2258-1G>A | / | / | NGS |
|  | *CHEK2* | Chr22:29091857 | c.1100delC | p.T367Mfs*15 | CD993415 | NGS |
| 3 | *BRCA2* | Chr13:32920978 | c.6952C>T | p.R2318* | CM993643 | NGS |
|  | *FANCM* | Chr14:45658326 | c.5101C>T | p.Q1701* | CM147953 | NGS |
| 4 | *BRCA2* | Chr13:32907419-32907420 | c.1804_1805del(G)2insG | p.G602Efs*12 | CD113845 | NGS |
|  | *RAD50* | Chr5:131930560 | c.1794-1G>T | / | / | NGS |
| 5 | *BRCA2* | Chr13:32912338-32912339 | c.3846_3847delTG | p.V1283Kfs*2 | CD961852 | NGS |
|  | *FANCM* | / | c.5717-?_6147+? | p.(G1906_I2048delins46) | / | aCGH, CNV-tool |
| 6 | *BRCA2* | Chr13:32914438 | c.5946delT | p.S1982Rfs*22 | CD961857 | NGS |
|  | *BUB1B* | Chr15:40512778 | c.2971G>T | p.E991* | / | NGS |
| 7 | *BRCA1* | Chr17:41209080-41209082 | c.5264_5266del(C)3ins(C)4 | p.Q1756Pfs*74 | CI941841 | NGS |
|  | *XRCC2* | Chr7:152357811-152357813 | c.94_96del(T)3ins(T)2 | p.F32Lfs*30 | CD159809 | NGS |
| 8 | *BRCA1* | Chr17:41243844-41243853 | c.3695_3704del(GTAAA)2insGTAAA | p.V1234Qfs*8 | CD104365 | NGS |
|  | *NBN* | Chr8:90983442-90983446 | c.657_661delACAAA | p.K219Nfs*16 | CD982819 | NGS |
| 9 | *BRCA1* | Chr17:41244280 | c.3268C>T | p.Q1090* | CM002244 | NGS |
|  | *BRCA2* | Chr13:32914759-32914761 | c.6267_6269delGCAinsC | p.Glu2089Aspfs*2 | CX972730 | NGS |

Legend to table S2: In the cohort of 523 OC patients, 114 different mutations were identified in a total of 146 patients. 9 out of these 146 patients carried two deleterious heterozygous germline variants. The variants identified in these 9 patients are listed. For each variant, the consequences on nucleotide and protein level according to the HGVS nomenclature (a, b), the HGMD accession number (c), detection method (d) are given.
